# Estimation of *C. elegans* cell- and tissue volumes

**DOI:** 10.17912/micropub.biology.000345

**Published:** 2021-01-04

**Authors:** Jonathan J Froehlich, Nikolaus Rajewsky, Collin Y Ewald

**Affiliations:** 1 Systems Biology of Gene Regulatory Elements, Berlin Institute for Medical Systems Biology, Max Delbrück Center for Molecular Medicine in the Helmholtz Association, Hannoversche Str 28, 10115 Berlin, Germany; 2 Eidgenössische Technische Hochschule Zürich, Department of Health Sciences and Technology, Institute of Translational Medicine, 8603 Schwerzenbach-Zürich, Switzerland

## Abstract

Although *C. elegans* is one of the best-studied model organisms, an estimate of its cell sizes and tissues is missing. Here we used the Virtual Worm that is based on electron microscopy images to calculate a zeroth-order approximation of cell and tissue sizes of *C. elegans*. We conclude that the intestine is the largest tissue, followed by the hypodermis, gonads, body wall muscles, pharynx, and neurons. Thus, we provide an approximation of tissue volumes of young adult *C. elegans*.

**Figure 1. Quantification of C. elegans’ cell and tissue volumes:**
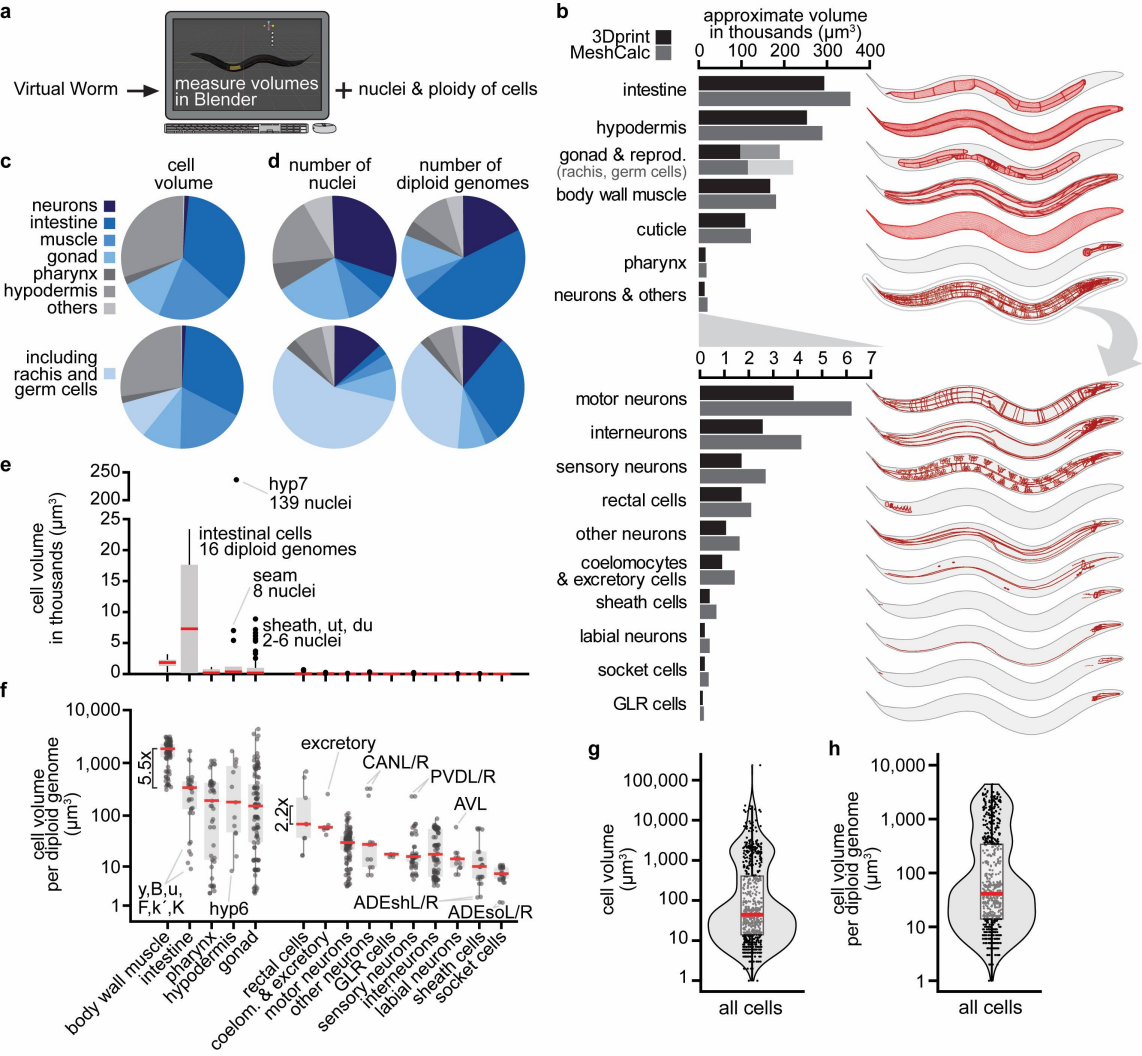
a) Workflow of our approach. b) On the left: tissue volumes determined with the two Blender plugins “3D print” or “Mesh Calculator”. For the gonad and reproductive system, the volume comprising the rachis and germ cells filling it in a lighter shade to distinguish them from somatic tissue. On the right: visualization of the analyzed tissues. c) Top: relative contribution of tissues to the final volume. The extracellular cuticle was excluded from this analysis. Bottom: same analysis but including rachis and non-somatic germ cells. d) Top: relative contribution of tissues to the number of nuclei, the number of diploid genomes. Bottom: same analysis but including the non-somatic germ cells. e) Cell volume, grouped by different tissues, displayed in a linear y-axis. Rachis and non-somatic germ cells were excluded from the analysis. f) Cell volume normalized per diploid genome, grouped by different tissues, displayed in a log_10_ y-axis. Rachis and non-somatic germ cells were excluded from the analysis. g) Distribution of cell volumes (log_10_ y-axis). h) Distribution of cell volumes per diploid genomes (log_10_ y-axis). For details, raw data, and calculations, please see Extended Data.

## Description

How large are *C. elegans’* tissues? To our surprise, we could not find any quantification of *C. elegans’* tissue volumes. By reaching out to the community, we received the following suggestions on how to quantify tissue volumes: First, using tissue-specific fluorescent reporter transgenes, confocal microscopy, and automated segmentation. Second and third, using electron microscopy serial sections (Altun *et al.* 2020) or methylene blue/pararosaniline-stained cross-sections (McGee *et al.* 2011) and manual or semi-automated segmentation. Here, we decided to use an alternative approach by directly measuring cell and tissue volumes from the Virtual Worm model (Grove and Sternberg 2011, http://caltech.wormbase.org/virtualworm/). The Virtual Worm is a three-dimensional model of a young adult *C. elegans* containing cell shape and position based on various sources, including the WormAtlas (Altun *et al.* 2020), the “Mind of the Worm” (White *et al.* 1986), and the “*C. elegans* Atlas” book (Hall and Altun 2007). We determined tissue volumes in the free software Blender by measuring individual cell volumes using the plugin “3D print toolbox” and directly with the plugin “Mesh volume calculator” (see Methods) (Figure 1a). Our quantifications revealed that the intestine was the largest tissue, followed by the hypodermis, gonads (including the volume of the rachis containing the non-somatic germ cells), body wall muscles, cuticle, pharynx, and neurons (Figure 1b, Extended Data). The majority of the total model volume was contributed by the four tissues intestine, hypodermis, body wall muscle, and gonads (including the rachis and germ cells), and each of these roughly contributed by a quarter (Figure 1c, bottom). However, several non-cellular structures were not included in these measurements, for example, the pericellular pseudocoelom, additional spaces in the uterus, and especially the large extracellular cuticle that together could take up 1/6^th^ or more of a total animal. The original Virtual Worm was based on resources that mostly relied on electron microscopy and dehydrating fixation protocols that reduce volume. This likely leads to a general underestimation of volumes. This is also indicated by a comparison between the total model volume of 1.4 million μm^3^ and the 2.2 million μm^3^ estimated for a live adult hermaphrodite (Grove 2012). Taken together, our calculations are a zeroth-order approximation of tissue size, however, we can infer relative tissue sizes. Thus, we conclude that intestine > hypodermis > gonads > body wall muscles > pharynx > neurons.

*C. elegans* contain several syncytial cells, for example, the pharyngeal pm1 cell (6 nuclei) or the hypodermal hyp7 cell (139 nuclei). Additionally, intestinal cells are polyploid and contain 16 diploid genomes (“32C”) at the young adult stage. We collected this information from the literature for all cells (Figure 1d, Extended Data). This allowed us to make several observations. For example, roughly one-third of somatic nuclei were contributed by neurons (Figure 1d, top, dark blue), but neurons minimally contributed to the final volume, with the caution that their volume might be underestimated due to the difficulty of modeling fine axonal structures (Figure 1c, top, dark blue). Germ cell nuclei are tightly packed, and although they accounted for more than half of all nuclei (Figure 1d, bottom, light blue), they took up only 1/8^th^ of the animal’s volume (Figure 1c, bottom, light blue).

We then analyzed individual cell volumes. The largest cell was the syncytial hypodermal hyp7, more than 10x larger than the second-largest cell, the polyploid intestinal int5L/R (Figure 1e). Intestinal cells were the largest cells on average (Figure 1e). To account for multiple genomic copies per cell (in syncytial- and polyploid cells), we normalized the cell volumes by the number of diploid genomes. This revealed that, on average, body wall muscle cells have a more than 5x larger volume per genomic copy than other cells (Figure 1f). mRNA and protein synthesis or their turnover might differ in these cells to support a larger cell volume with the same genomic copies. Additional observations could be made from the data. For instance, tissues that contributed most to the final animal volume also contained larger cells on average (see body wall muscle, intestine, hypodermis, and gonads in Figure 1f). The cell volume per diploid genome for 86% of cells was below 1,000 μm^3^, and for 70% of cells even below 200 μm^3^ (Figure 1h). Absolute estimates for single cells might be underestimated, as already noted above. Their comparison also has to be taken with caution because their modeling does not contain fine morphological structures.

In conclusion, we provide an approximation of tissue volumes of young adult *C. elegans* based on the Virtual Worm project. These values are rough estimates, and future experiments could determine precise volumes and variability between animals. Our results might be useful for interpreting relative tissue contributions of whole *C. elegans* for transcriptomics, proteomics, epigenomics, or when performing gene-specific detection by qRT-PCR and Western blot. The approximations of cell volumes and their relationship to genomic copies might be useful for the planning and interpretation of single-cell/single-nuclei methods in *C. elegans* and related nematodes.

## Methods

We determined the volumes of each cell from the Virtual Worm (file retrieved at: http://canopus.caltech.edu/virtualworm/Virtual%20Worm%20Blend%20File/Virtual_Worm_February_2012.blend) (Grove and Sternberg 2011) using the free 3D modeling and rendering software Blender (version 2.90). For this, we used the default addon “3D print toolbox”, which comes bundled with Blender 2.90. We manually selected each cell and wrote down the volume. We then calculated volume by tissue from these data. Separately, we also directly measured volumes of tissues (grouped as “collections” in the Virtual Worm Blender file) using the small addon “Mesh Volume Calculator” (available at https://blendermarket.com/products/mesh-volume-calculator). Mesh Volume Calculator exports the volume of each collection (representing tissues) automatically to a csv. We first applied the Blender operation “scale” to all objects in the model and then used Mesh Volume Calculator to export the volumes. We also slightly re-grouped the tissues, mainly to separate cuticle from hypodermis and rachis/germ cells from the somatic gonads. To estimate volumes in μm^3^ from Blender volumes, we assumed that the “100 µm cube” object corresponded to 100 x 100 x 100 = 1,000,000 μm^3^. To verify the correct order of magnitude, we compared our approximations of total model volume (1.4 million μm^3^) to the volume estimated for a live adult hermaphrodite (2.2 million μm^3^) obtained from https://wiki.wormbase.org/index.php/Worm_numbers.

The number of nuclei per cell was obtained from Wormatlas (Altun *et al.* 2020) and using the Ontology browser at Wormbase (Harris *et al.* 2020) at http://www.wormbase.org/tools/ontology_browser using the nodes: “Anatomy Ontology”>”*C. elegans* anatomical entity”>”functional system”>”organ system”>. Alternatively, we directly accessed the anatomical ontology at http://www.ontobee.org/ontology/WBbt?iri=http://purl.obolibrary.org/obo/WBbt_0000100. Information about syncytial cells can also be found directly at http://www.ontobee.org/ontology/catalog/WBbt?iri=http://purl.obolibrary.org/obo/WBbt_0008074. We double-checked information on syncytial cells using Table 1 in Shemer and Podbilewicz 2000. Approximate germ cell numbers were obtained for sperm from Singson 2001 and for germ cells from Diag and Schilling *et al.* 2018.

Schematic representations of tissues (Figure 1b) were produced by adjusting the view in Blender, selecting the tissue, performing a screenshot, and converting the screenshot into vector graphics in Adobe Illustrator using the “trace” function. For this, the lateral perspective was chosen in Blender and the following settings were used: Background=”World”. Viewport shading=”solid mode” or “as wire edges”. Only the selected tissue was displayed. In solid mode the wireframe option was turned off, resulting in an image with only cell outlines. For displaying as wire edges, the wireframe was shown, slightly indicating the three-dimensional shape of the selected objects. For this latter option, several “angle thresholds” were tried between 0.5-1, to set the number of displayed mesh lines.

## Reagents

**Data availability**

Extended data are archived and available on Caltech Data: 10.22002/D1.1835.

**Extended Data.** Cell- and Tissue Volumes, including Syncytia and Ploidy. This table provides all the primary data obtained from measuring cell and tissue volumes of the Virtual Worm and the information collected from the literature on ploidy and syncytia.
