# Corrigendum: Estimation of *C. elegans* cell- and tissue volumes

**DOI:** 10.17912/micropub.biology.000552

**Published:** 2022-04-09

## Description

Due to human error the Extended Data Table was missing the measurements for the MSNL/R cells and contained wrong measurements for OLQDL/R cells.Correct measurements are NSML: 12 μm^3, NSMR: 12 μm^3, OLQDL: 19 μm^3, OLQDR: 19 μm^3.We corrected this in a new version of the Extended Data Table.These changes do not affect the main analyses and plots because they lie below the relevant order of magnitudes.

The updated dataset can be accessed at https://data.caltech.edu/records/20080.

